# Discussing prognosis with patients with osteoarthritis: a cross-sectional survey in general practice

**DOI:** 10.1007/s10067-015-3094-8

**Published:** 2015-10-16

**Authors:** L. E. Clarson, B. I. Nicholl, A. Bishop, R. Daniel, C. D. Mallen

**Affiliations:** Research Institute for Primary Care & Health Sciences, Arthritis Research UK Primary Care Centre, Keele University, Keele, Staffordshire ST5 5BG UK; General Practice & Primary Care, Institute of Health and Wellbeing, University of Glasgow, Glasgow, UK

**Keywords:** Osteoarthritis, Primary care, Prognosis

## Abstract

Osteoarthritis is a leading cause of chronic pain and disability and one of the most common conditions diagnosed and managed in primary care. Despite the evidence that patients would value discussions about the course of osteoarthritis to help them make informed treatment decisions and plan for the future, little is known of GPs’ practice of, or views regarding, discussing prognosis with these patients. A cross-sectional postal survey asked 2500 randomly selected UK GPs their views on discussing prognosis with patients with osteoarthritis and potential barriers or facilitators to such discussions. They were also asked if prognostic discussions were part of their current practice and what indicators they considered important in assessing the prognosis associated with osteoarthritis. Of 768 respondents (response rate 30.7 %), the majority felt it necessary to discuss prognosis with osteoarthritis patients (*n* = 738, 96.1 %), but only two thirds reported that it was part of their routine practice (*n* = 498, 64.8 %). Most respondents found predicting the course of osteoarthritis (*n* = 703, 91.8 %) and determining the prognosis of patients difficult (*n* = 589, 76.7 %). Obesity, level of physical disability and pain severity were considered the most important prognostic indicators in osteoarthritis. Although GPs consider prognostic discussions necessary for patients with osteoarthritis, few prioritise these discussions. Lack of time and perceived difficulties in predicting the disease course and determining prognosis for patients with osteoarthritis may be barriers to engaging in prognostic discussions. Further research is required to identify ways to assist GPs making prognostic predictions for patients with osteoarthritis and facilitate engagement in these discussions.

## Introduction

Osteoarthritis (OA) is the most common form of arthritis worldwide, with an estimated prevalence of 20–30 % [1]. It has come to be recognised as a chronic condition associated with increased morbidity and mortality [1, 2]. The Global Burden of Health Survey (2010) reported OA to be responsible for 17.1 million years lived with disability worldwide and to be the fastest growing chronic disease [2]. OA is one of the most common diagnoses made in general practice, the setting where the majority of patients are managed. Currently, the medical focus of OA treatment is symptom control. Whilst symptom relief is important, it is not the only component of chronic disease management that is important to patients; 80 % of older people presenting to their GP with musculoskeletal pain reported discussion of the likely course and outcome (prognosis) of their condition was important to them, in order to help them make decisions and plan for the future [3].

Prognostic discussion is most commonly associated with survival in life-limiting illnesses such as cancer; however, patient-centred prognostic outcomes such as symptom severity, functional status and health-related quality of life are increasingly being investigated in the context of other common non-life-threatening conditions [4]. Physicians frequently report that they feel poorly prepared for prognostication, finding it stressful and difficult to make accurate prognostic predictions and that they fear adverse patient opinion that may result from inaccurate prognostic discussions [5]. Prognostic error is common and may have adverse effects on both patient care and social policy [6], and so it is important that the role of prognostic discussions in chronic disease management is understood and optimised to minimise adverse effects.

Only one small study has investigated the views of GPs (all of whom had an interest in rheumatology) on prognostic discussions with patients with OA [7], despite evidence that patients value prognostic information [3, 8]. We sought to build on this by conducting a larger survey investigating GPs’ views on, and practice of, discussing prognosis with patients with OA and factors which they feel facilitate or prevent these discussions.

## Methods

### Ethical approval

The study was approved by the North Staffordshire Local Research Ethics Committee (09/H1204/65).

### Participants

A random sample of 2500 practicing UK GPs was sent an eight page self-completion postal questionnaire. The sample was provided by the Binley’s database, a for-profit organisation providing health professionals’ contact details. Potential participants were selected from their database at random. Non-responders were mailed a reminder postcard after 2 weeks and a further copy of the questionnaire 2 weeks later.

### Questionnaire design

The questionnaire used in this study was adapted from a survey of GPs views on discussing prognosis in patients with COPD [9]. A musculoskeletal version was developed and originally administered at a Primary Care Rheumatology workshop to GPs with a special interest in musculoskeletal medicine [7]. It was subsequently refined and piloted at two general practices and during a local teaching session for GP registrars in order to ensure that the questions were easy to read and not ambiguous, and that it could be completed in less than 15 minutes.

### Questionnaire content

Respondents provided demographic details, including gender, job title, and whether they had a special interest in musculoskeletal medicine or additional post-graduate qualifications. They were then asked a series of questions about prognostic discussions. These are detailed in Box 1.

**Table:** 

Box 1: Views on, and practice of, discussing prognosis in patients with OA
• How necessary do you consider discussion of prognosis when treating patients with cancer, chronic obstructive pulmonary disease (COPD), diabetes, ischaemic heart disease (IHD), heart failure (HF), epilepsy and OA? • How frequently do you discuss prognosis with patients with those conditions? • How often should prognosis be discussed with patients with OA? • When in the course of OA should prognosis be discussed? • Who is responsible for initiating such discussions? • Do you prioritise prognostic discussions during OA consultations? • Do you find it difficult to make a prognosis or predict the course of OA? • What are the five most important prognostic indicators in OA? • Do you screen patients with OA for anxiety and depression?
Barriers and facilitators to prognostic discussions in OA
• Which patient factors (e.g., current employment or active lifestyle) affect the likelihood of you discussing prognosis with OA patients? • Which outcomes may result from prognostic discussions? (e.g. patients may find it upsetting) • What are the potential barriers to deciding to discuss prognosis with OA patients? (e.g. time constraints)

Copies of the questionnaire are available from the lead author on request.

### Statistical analysis

The results were analysed using PASW Statistics 21 (release 21.0.0). Data were first analysed descriptively and associations between key variables were subsequently explored using chi-squared tests. Respondents with some missing data were included but only the complete data for individual questions were analysed.

## Results

Of the 2500 questionnaires sent, 768 were returned completed (30.7 %). Overall, 541 (70.4 %) respondents were male and 684 (89.1 %) were principals within their practices. Characteristics of respondents are summarised in Table 1.

**Table 1:** Demographic characteristics of respondents

Characteristics	Respondents to the survey n (%)^a^
Gender	
Male	541 (70.4)
Female	223 (29.0)
Missing	4 (0.5)
Job title	
Partner	684 (89.1)
Salaried	29 (3.8)
Missing	55 (7.2)
Special Interest in MSM	176 (22.9)
Size of practice (number of registered patients)	
Small (<4000)	183 (23.8)
Medium (4000–7999)	294 (38.3)
Large (>8000)	291 (37.9)
Missing	0 (0)

*MSM* musculoskeletal medicine

^a^Total may not equal 100 due to rounding

### Views on, and practice of, discussing prognosis with patients with OA

**Fig. 1:**
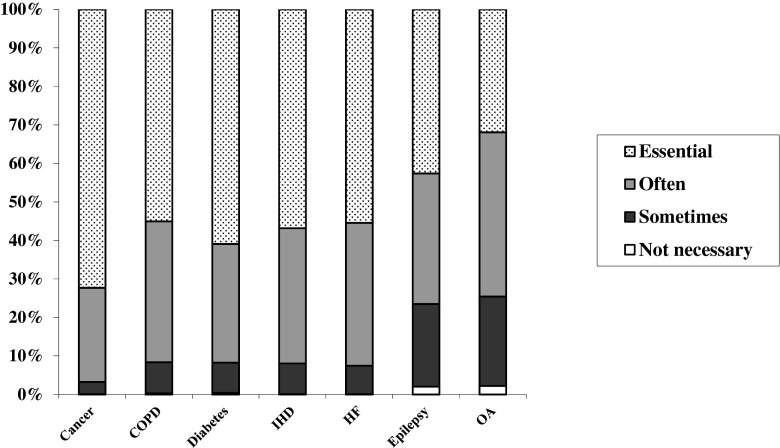
Necessity of prognostic discussion

GPs reported it necessary to have prognostic discussions, with patients with cancer and diabetes, but least so with patients with OA (Fig. 1).

**Fig. 2:**
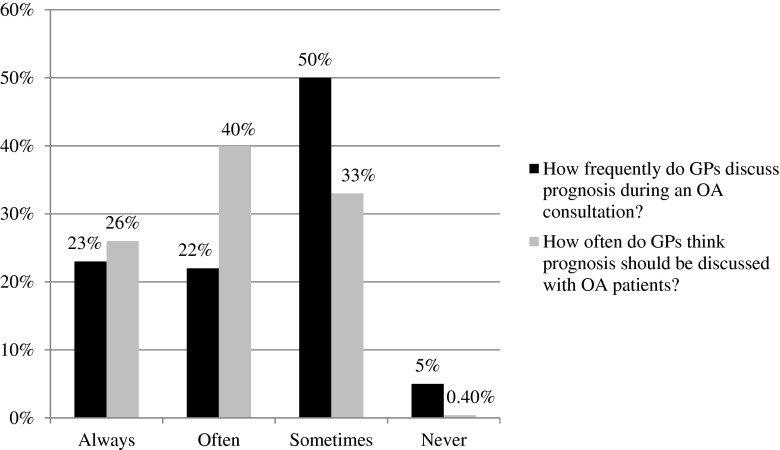
Frequency of prognostic discussion

Similar proportions often consider it necessary to discuss prognosis with patients with OA (*n* = 322/41.9 %), and consider that this should be done often as part of routine care (*n* = 301/39.2 %), yet only just over half of those are able to actually deliver this in practice (*n* = 167/21.7 %). Most GPs reported having prognostic discussions with patients with OA “sometimes” (*n* = 376/49.0 %), whereas only 246 (32.0 %) indicated this should be the frequency of prognostic discussion. Similar proportions of GPs favoured always discussing prognosis with OA patients (*n* = 197/25.7 %) and also reported doing so (*n* = 169/22.0 %). Whilst only three respondents (0.4 %) thought that prognosis should never be discussed with patients with OA, more (*n* = 39/4.7 %) responded that they “never” actually had such discussions (Fig. 2).

Most GPs felt that responsibility for initiating prognostic discussions with patients with OA lay with them (*n* = 667/86.8 %), but more than half felt that patients should also be responsible for initiating these discussions (*n* = 441/57.4 %). GPs were most likely to discuss prognosis at the request of the patient (*n* = 197/25.7 %), when treatment changed (*n* = 149/19.4 %), or at the first visit (*n* = 142/18.5 %), but this was only prioritised within the OA consultation by *n* = 90 (11.7 %).

**Fig. 3:**
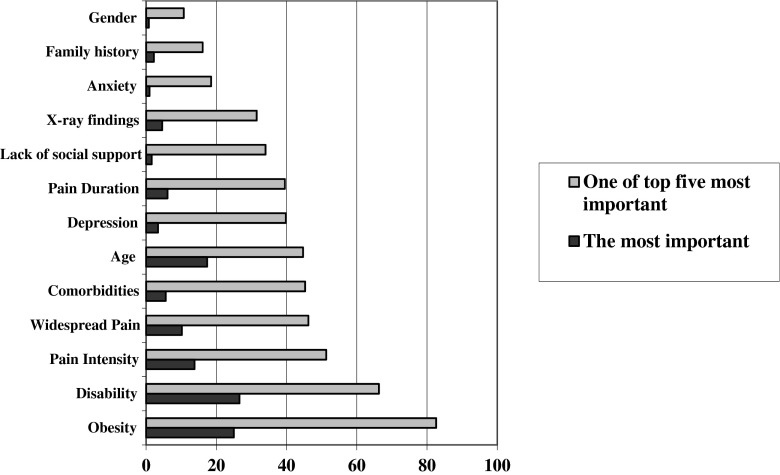
Respondents views on the most important prognostic indicators in OA

The prognostic indicators most commonly reported by respondents are illustrated in Fig. 3. Disability was considered to be the strongest prognostic indicator in patients with OA, ranked number 1 (most important) by 204 (26.6 %) of respondents, followed by obesity (*n* = 192/25.0 %). Obesity was ranked as one of the five most important prognostic indicators by the greatest proportion (*n* = 634/82.6 %), followed by disability (*n* = 509/66.3 %), whilst gender, family history and anxiety were included least often.

Only a minority of respondents reported screening OA patients for depression (*n* = 196/26 %) or anxiety (*n* = 140/18 %).

### Barriers and facilitators to prognostic discussions in OA

The majority of GPs reported some difficulty in determining prognosis (*n* = 589/76.7 %) or predicting the course of OA (*n* = 705/91.8 %).

When asked about patient characteristics, GPs reported they were more likely to discuss prognosis with patients with an active lifestyle (*n* = 511/66.5 %), pre-existing knowledge of OA (*n* = 487/63.4 %) and those in current employment (*n* = 459/59.8 %). The majority felt that discussing prognosis with patients with OA would have a beneficial effect by improving self-management (*n* = 659/85.8 %) and by making patient expectations more realistic (*n* = 620/80.7 %), rather than a detrimental one by removing hope (*n* = 120/15.6 %).

Time constraints were the most frequently reported barrier to prognostic discussions in OA (*n* = 440/57.3 %), followed by lack of training (*n* = 138/18.0 %) and waiting for the patient to ask (*n* = 96; 12.5 %).

### GP characteristics associated with engaging in prognostic discussions in OA

None of the demographic characteristics studied were associated with responses regarding necessity or frequency of prognostication for patients with OA. However, more female respondents reported that they found making a prognosis in patients with OA difficult (50 %:34 %; *χ*^2^ = 16.83 *p* < 0.01), although both genders reported similar difficulty in predicting the course of OA.

Having a special interest in musculoskeletal medicine (MSM) or having read the NICE OA guidelines was associated with several aspects of engaging in prognostic discussions (summarised in Table 2). These respondents were more likely to consider prognostic discussions often necessary or essential than those without the additional training or knowledge, and to discuss prognosis with patients with OA more frequently. They were also less likely to report difficulty in making a prognostic prediction or predicting the course of OA.

**Table 2:** Associations with engaging in prognostic discussions

Concerning patients with OA…	Special interest in MSM?			Having read the NICE OA guidelines?		
	Yes %	No %	*χ* ^2^ (p for significance)	Yes %	No %	*χ* ^2^ (*p* for significance)
Prognostic discussions are often necessary or essential	79.9	72.9	12.6 (<0.01)	78.5	69.3	20.5 (<0.01)
I think prognosis should be discussed often or always	74.3	64.4	18.0 (<0.01)	72.2	59.2	15.3 (<0.01)
I do discuss prognosis often or always	47.1	44.3	18.4 (<0.01)	49.5	38.8	21.2 (<0.01)
Prognosis should be discussed when treatment changes	35.2	26.1	9.8 (0.02)	30.9	24.6	9.9 (0.02)
Prognosis should only be discussed at the patient’s request	26.4	41.1	9.8 (0.02)	31.9	45.1	9.9 (0.02)
I prioritise prognostic discussions	19	10	13.3 (0.01)	13.9	9.7	20.1 (<0.01)
It is difficult to predict the course of OA	26.6	43.3	16.7 (<0.01)	62.9	65.0	0.97 (0.62)
It is difficult to make a prognosis for OA	55.7	66.7	7.1 (0.03)	35.3	43.6	9.0 (0.01)

*MSM* musculoskeletal medicine, *OA* osteoarthritis

GPs with a special interest in MSM were less likely to be influenced by patient factors such as age (49.1 %:56.0 % *p* < 0.01), pre-existing patient knowledge (66.8 %:61.6 % *p* = 0.03), co-existing medical conditions (53.1 %:56.3 % *p* < 0.01) and patients being employed (55.7 %:63.0 % *p* = 0.03) in their decision to discuss prognosis with patients with OA than those without.

## Discussion

These findings suggest that GPs consider prognostic discussions necessary in patients with OA, favour frequent discussion of prognosis and feel that it is the responsibility of both GPs and patients to initiate such discussions. Despite this, only a minority of respondents prioritised discussion of prognosis during an OA consultation, with the majority reporting difficulty in predicting the course of OA and making a prognosis for OA patients.

Our findings demonstrate a clear difference between GPs’ views on the importance of discussing prognosis with patients with OA and their practice of doing it. A similar mismatch has been reported in the attitudes of GPs to discussing prognosis with both COPD and cancer patients, with reasons cited including lack of confidence in the ability to prognosticate due to uncertainty about timeframes for progression of the disease, and concern about loss of patient confidence that may result from an inaccurate prognosis [5, 10].

The top 5 prognostic indicators preferred by respondents (obesity, disability, pain intensity, widespread pain and co-morbidity) reflect a mixture of evidence-based predictors of disease (e.g. obesity) [11], and patient reported measures, such as disability and pain intensity. This may result from an underlying belief that it is these more subjective measures which give the greatest insights into individual disease progression, or simply represent attempts at personalised prognostication using what is known about the patient in the absence of agreement on the course of OA or a reliable set of prognostic variables.

Existing literature about predictors of OA progression is limited, conflicting or inconclusive. A recent systematic review suggests that age, ethnicity, BMI, co-morbidity count and baseline severity are associated with progression of clinical knee OA [12]; however, it reports the lack of agreement between studies about what constitutes disease progression, and the paucity of studies which measure symptom progression as an outcome as an important limitation, preventing meta-analysis and limiting the applicability of these findings to patients, in whom pain has been reported as a primary concern [13]. It would therefore seem vital that more studies define disease progression in terms that are relevant to prognostic discussions with patients.

It is becoming accepted that prognosis is influenced by a complex array of biological, clinical and social factors [14], and it is, therefore, perhaps not surprising that our findings support the evidence that doctors find prognostication in general difficult [15]. Heterogeneity of OA pathology, fluctuation of pain and physical limitation due to OA over time, and a lack of consensus on measures of progression and endpoint definition (both structural and clinical), make OA prognostication particularly difficult [12, 16]. It is possible that the difference between thinking prognostic discussions are important, and engaging in them, is the recognition that although important to patients, prognostication in OA is attempting to predict the unpredictable.

The clinical implications of these findings are that whilst GPs feel discussing prognosis is an important part of OA care, they find it difficult due to the lack of time, and the challenge of correctly predicting the course of the disease. Whilst specialist training in MSM is perhaps beyond the requirements of most GPs, simply reading the NICE OA guidelines also facilitated clinician’s engagement and confidence in prognostication. It is possible that improved targeted dissemination of such guidance to GPs may be an effective way to remove some of the barriers to discussing prognosis in OA. It may be that it is unrealistic to ask clinicians to predict the prognosis of a disease whose course is so highly individualised and influenced by both medically and socially complex factors, particularly given the lack of agreement on the characteristics which influence prognosis. However, it could also be argued that the added importance of discussing prognosis with OA patients is to highlight the influence of modifiable and social factors, such as obesity, on disease experience and thus prognosis. Respondents would seem to agree with this since the majority agreed that prognostic discussions could make patient expectations more realistic and improve self-management. Further research is needed to better understand the measures of prognosis that are most important to patients and identify the characteristics which influence these, in order to better equip physicians to undertake accurate prognostication that is meaningful to patients.

There are a number of strengths and limitations to this study. This is the first large scale study of its kind to explore the views of a random sample of UK GPs on discussing prognosis with OA patients. The findings mirror those of the previous smaller study suggesting accuracy of the results. The adaptation of a previously used questionnaire and piloting with a sample of GPs strengthens the reliability of our findings. Questionnaire studies of GPs typically have low response rates, and whilst ours is no exception, it is in line with previously published studies [17]. Comparison of respondents with the UK population is difficult due to a paucity of available information. The majority of respondents were male (70 %), and GP principals (89 %), and this generally reflects the GP population in England (52 % male; 68 % GP principals) [18]. Female GPs and non-principal (e.g. salaried, locum and sessional) GPs appear to be underrepresented in our sample. Whilst no data about gender were held for non-respondents, the majority of non-respondents were also GP principals, and so response bias cannot necessarily be inferred. However, non-principal GPs as a more mobile population are both less likely to have longstanding relationships with patients resulting in the need for prognostic discussions, and more difficult to contact in a postal survey of this nature. As a result, it is possible that they may have different views about discussing prognosis in chronic disease that are not represented in these findings.

## Conclusion

Although GPs consider prognostic discussions necessary in patients with OA, few prioritise discussion of prognosis during an OA consultation. Lack of time and perceived difficulties in predicting the course of OA and making a prognosis for patients with OA may be barriers to engaging in prognostic discussions. GPs with a special interest in MSM and those who have read the NICE OA guidelines are more likely to engage in prognostication. Interventions to assist GPs in making a prognosis for patients with OA would be a valuable step in improving the management of OA in patients with this common condition.
